# Persistent fatigue induced by interferon-alpha: a novel, inflammation-based, proxy model of chronic fatigue syndrome

**DOI:** 10.1016/j.psyneuen.2018.11.032

**Published:** 2019-02

**Authors:** Alice Russell, Nilay Hepgul, Naghmeh Nikkheslat, Alessandra Borsini, Zuzanna Zajkowska, Natalie Moll, Daniel Forton, Kosh Agarwal, Trudie Chalder, Valeria Mondelli, Matthew Hotopf, Anthony Cleare, Gabrielle Murphy, Graham Foster, Terry Wong, Gregor A. Schütze, Markus J. Schwarz, Neil Harrison, Patricia A. Zunszain, Carmine M. Pariante

**Affiliations:** a Dept. of Psychological Medicine, Institute of Psychiatry, Psychology and Neuroscience, King’s College London, UK; b Institute of Laboratory Medicine, University Hospital, LMU Munich, Munich, Germany; c Gastroenterology & Hepatology Department, St George’s University Hospitals NHS Foundation Trust, London, UK; d Institute of Liver Studies, Kings College Hospital NHS Foundation Trust, London, UK; e Chronic Fatigue Service, South London and Maudsley NHS Foundation Trust, Maudsley Hospital, London, UK; f The Royal Free London Fatigue Service, Royal Free London NHS Foundation Trust, London, UK; g Gastrointestinal and Liver services Department, Barts Health NHS Trust, London, UK; h Gastroenterology & Hepatology Department, Guy’s & St Thomas’ NHS Foundation Trust, St Thomas’ Hospital, London, UK; i Brighton and Sussex Medical School, University of Sussex, Brighton, UK

**Keywords:** Chronic fatigue syndrome, Inflammation, Fatigue, Cytokines, Kynurenine, Tryptophan

## Abstract

The role of immune or infective triggers in the pathogenesis of Chronic Fatigue Syndrome (CFS) is not yet fully understood. Barriers to obtaining immune measures at baseline (i.e., before the trigger) in CFS and post-infective fatigue model cohorts have prevented the study of pre-existing immune dysfunction and subsequent immune changes in response to the trigger.

This study presents interferon-alpha (IFN-α)-induced persistent fatigue as a model of CFS. IFN-α, which is used in the treatment of chronic Hepatitis C Virus (HCV) infection, induces a persistent fatigue in some individuals, which does not abate post-treatment, that is, once there is no longer immune activation. This model allows for the assessment of patients before and during exposure to the immune trigger, and afterwards when the original trigger is no longer present.

Fifty-five patients undergoing IFN-α treatment for chronic HCV were assessed at baseline, during the 6–12 months of IFN-α treatment, and at six-months post-treatment. Measures of fatigue, cytokines and kynurenine pathway metabolites were obtained. Fifty-four CFS patients and 57 healthy volunteers completed the same measures at a one-off assessment, which were compared with post-treatment follow-up measures from the HCV patients.

Eighteen patients undergoing IFN-α treatment (33%) were subsequently defined as having ‘persistent fatigue’ (the proposed model for CFS), if their levels of fatigue were higher six-months post-treatment than at baseline; the other 67% were considered ‘resolved fatigue’. Patients who went on to develop persistent fatigue experienced a greater increase in fatigue symptoms over the first four weeks of IFN-α, compared with patients who did not (Δ Treatment Week (TW)-0 vs. TW4; PF: 7.1 ± 1.5 vs. RF: 4.0 ± 0.8, *p* = 0.046). Moreover, there was a trend towards increased baseline interleukin (IL)-6, and significantly higher baseline IL-10 levels, as well as higher levels of these cytokines in response to IFN-α treatment, alongside concurrent increases in fatigue. Levels increased to more than double those of the other patients by Treatment Week (TW)4 (*p* =  0.011 for IL-6 and *p* = 0.001 for IL-10). There was no evidence of an association between persistent fatigue and peripheral inflammation six-months post-treatment, nor did we observe peripheral inflammation in the CFS cohort. While there were changes in kynurenine metabolites in response to IFN-α, there was no association with persistent fatigue. CFS patients had lower levels of the ratio of kynurenine to tryptophan and 3-hydroxykynurenine than controls.

Future studies are needed to elucidate the mechanisms behind the initial exaggerated response of the immune system in those who go on to experience persistent fatigue even if the immune trigger is no longer present, and the change from acute to chronic fatigue in the absence of continued peripheral immune activation.

## Highlights

• Baseline fatigue is not associated with the development of persistent fatigue after IFN-α.
• IFN-α-induced persistent fatigue is associated with increased baseline IL-10.
• Patients who develop persistent fatigue experience greater increases in IL-6 and 10 in response to IFN-α.
• Persistently fatigued patients recover at a similar rate, but from a more severe acute response to the initial trigger.
• Once established, neither the persistent fatigue phenotype, nor CFS, are associated with peripheral immune activation.

## 1 Introduction

There is some evidence implicating the immune system in the pathogenesis of Chronic Fatigue Syndrome (CFS), but the exact role of immune mechanisms in this condition, especially at its onset, have yet to be established. Genetic polymorphisms in immune genes are associated with (CFS) as well as other disease-related fatigue (Wang et al., 2017). Some recent studies have observed raised inflammatory markers in CFS patients, both in serum and cerebrospinal fluid (Maes et al., 2012; Silverman et al., 2010). Increased levels of the inflammatory biomarker, C-reactive protein, have been described in one of the largest CFS studies to date (Raison et al., 2009). Moreover, a recent study has found that 17 cytokines in the serum, mainly pro-inflammatory, correlate with Myalgic Encephalomyelitis (ME)/CFS severity (Montoya et al., 2017). However, a meta-analysis of findings published up to 2003 found no consistent evidence for immune dysfunction (Lyall et al., 2003); and a more recent meta-analysis found that, of the 77 markers measured, only transforming growth-factor beta (TGF-β) was consistently raised (Blundell et al., 2015), a finding also confirmed by the aforementioned study by Montoya et al. (2017).

To investigate the role of the immune system further, post-infective fatigue syndrome has been used as a model for CFS. Such studies have prospectively monitored new cases of infection and found clinical and socio-demographic factors, as well as genetic and blood immune markers that were associated with post-infective CFS (Candy et al., 2002; Hickie et al., 2006; Piraino et al., 2012; Vollmer-Conna et al., 2004). While this model has the advantage of assessing individuals at the onset of CFS, no study has been able to assess individuals *before and after* the infective trigger, due to the random occurrence of infections; thus we cannot establish whether pre-existing immune dysfunction characterises individuals at risk of post-infective CFS. The study of persistent fatigue induced by interferon-alpha (IFN-α), a novel model presented here, allows us to do just that.

IFN-α is administered for the treatment of Hepatitis C Virus (HCV) infection for courses of six months or more. The treatment induces acute sickness behaviours in the first few weeks, including myalgia, malaise, anorexia, fatigue, and mild cognitive impairment. Between one third and half of patients then continue to experience fatigue and cognitive symptoms, with additional mood disturbance usually occurring after eight to twelve weeks of treatment. These clinical and biological phenomena have been extensively characterised (Capuron et al., 2002; Capuron and Miller, 2004; Maddock et al., 2005). Though less well studied, patients have also reported *persistent fatigue six-months after cessation of IFN-α therapy*, with evidence from patients’ surveys that up to 60% of patients experience fatigue and other persistent CFS-like symptoms, including cognitive impairment and joint and muscle aches (Hepatitis and Trust, 2010; Hopwood, 2013). Interestingly, the original immune trigger (in this case, IFN-α) is no longer present at this stage, another similarity often noted in CFS. These considerations make IFN-α a suitable model to mimic the immune trajectory of people who develop CFS, at baseline (i.e., before the immune trigger), during the immune trigger, and after the immune trigger has ceased. This is the first study to investigate IFN-α-induced persistent fatigue in such detail, and we have examined immunological and psychosocial factors that predict this condition, in a prospective manner. We have also cross-sectionally compared these subjects with clinically defined CFS patients and healthy controls.

This study has explored possible predictors shown previously to be associated with CFS, CFS-like illnesses and/or IFN-α induced fatigue. In relation to psychosocial factors, childhood trauma has been identified as being a risk factor for CFS, as well as ‘CFS-like’ illnesses (Borsini et al., 2013; Clark et al., 2011; Heim et al., 2009, 2006), and, independently, for inflammation in adulthood (Danese et al., 2007). Experience of stressful life events generally may also contribute to the risk of CFS (Hatcher and House, 2003; Salit, 1997), though another study found no link with recent stressors in the last 12 months (White et al., 2001). A personal history of a psychiatric disorder has also been found to be a risk factor for CFS, with increased levels of psychopathological symptoms preceding the symptoms of fatigue (Clark et al., 2011; Harvey et al., 2008). Lastly, the limited studies that have assessed fatigue post-IFN-α treatment have found an association with treatment outcome, with those with treatment success (sustained viral response, i.e., treatment success shown as viral RNA undetected six-months post-treatment) experiencing greater fatigue (Huckans et al., 2015; Sarkar et al., 2012). All of these clinical variables have been assessed in the present study.

With regards to biological measures, in addition to the cytokine measurements referenced above, this study is, to the best of our knowledge, one of the first studies to examine a wide range of kynurenine pathway metabolites both in CFS patients, and in the context of persistent fatigue following an immune trigger. An imbalance in tryptophan metabolism as prompted by increases in inflammation has been implicated in the somewhat overlapping groups of symptoms that are relevant to CFS, spanning pain, fatigue and depression. For example, quinolinic acid (QUIN) has an excitotoxic effect on the glutamate N-methyl-d-aspartate (NMDA) receptor, the stimulation of which may cause hyperalgesia and central sensitisation, or an increased sensitivity to pain (Anderson et al., 2014; Romano et al., 2015). Of relevance, drugs that block the NMDA receptor have shown beneficial effects for the treatment of pain associated with central sensitisation, as has been seen in CFS and the associated condition, fibromyalgia (Nijs et al., 2011). In a study of chronic-activated Epstein-barr virus (CAEBV), higher fever was significantly associated with a higher kynurenine/tryptophan (Kyn/TRP) ratio and lower TRP, while there were trends towards associations between a higher KYN/TRP ratio and severe tiredness and night sweats (Bellmann-Weiler et al., 2008). This suggests that inflammation-induced activation of IDO may be relevant for the persistence of symptoms, including fatigue, following an immune trigger. The pathway has been extensively studied in depression and other neuropsychiatric diseases, as well as other medical conditions where immune activation and inflammation are key features, where increased ratios of kynurenine to tryptophan have also been found (Leonard and Maes, 2012; Morris et al., 2015). That said, the literature relating to CFS specifically is limited, and so this aspect of the approach can be considered exploratory.

## 2 Methods

The study was approved by the London Dulwich Research Ethics Committee (REC ref: 12/LO/1368).

### 2.1 Prospective study

We recruited 55 patients (mean age 44.6 ± 1.6 years; 80% male) from liver outpatient services at King’s College Hospital and five other hospitals in London. Eligible patients were adults with chronic HCV infection who were due to commence antiviral therapy with IFN-α and ribavirin. In addition to IFN-α/rib, eight subjects (14%) were also prescribed a direct-acting antiviral (‘triple therapy’, adding boceprevir, telaprevir or simeprevir), after treatment protocols changed across the UK. The sample is partially overlapping with that in the publication by Hepgul et al. (2016a, 2016b) (of *n* = 55, overlap of *n* = 35), though post-treatment data was not presented in Hepgul et al.’s paper. Exclusion criteria included any autoimmune disorders or causes of liver disease other than HCV, and co-infection with HIV or Hepatitis B. All but 3 patients were free from depression and antidepressants at baseline. Clinical assessments were conducted at baseline (treatment week 0; TW0), TW4, TW8 and TW12, and at the end of treatment (67% at TW24, the remaining by TW36 or TW48). This was followed by a further assessment six-months after the end of treatment, where the persistent fatigue phenotype was identified. Eighteen patients (33%) were defined as having ‘persistent fatigue’ (PF; the proposed model for CFS) if their levels of fatigue were higher at follow-up than at TW0 (measured using the Chalder Fatigue Questionnaire; see below); the other 67% were defined as having ‘resolved fatigue’ (RF).

### 2.2 Cross-sectional study

The cross-sectional study compared data at the six-month follow-up visit (that is, when the CFS-like phenotype of PF was identified) from the PF and the RF groups as well as patients with clinically-defined CFS and healthy volunteers (controls). We recruited *n* = 54 CFS patients from the CFS specialist clinics at the South London and Maudsley (SLaM) and the Royal Free NHS Foundation Trusts, diagnosed according to the Oxford criteria (Sharpe et al., 1991), and *n* = 57 healthy volunteers, free from recent or recurrent mental disorders, including past or present substance abuse or dependency. Healthy controls were recruited from a number of sources: advertisements placed on a classified adverts website (‘Gumtree’); a circular email sent out to all staff and students of King’s College London; leaflets placed in local community settings and through word of mouth. Participants in both groups were excluded if they had significant health conditions known to influence the immune system.

Consistent with the literature (Alter, 2007; Clark et al., 2011; Jason et al., 2003), CFS patients were younger than the HCV group and predominantly female (mean age 37.2 ± 1.5 years and 31% male in CFS vs. 44.6 ± 1.6 years and 80% male in HCV). Therefore, the controls were selected to be comparable to the larger group of HCV and CFS patients combined (mean age 40.8 ± 1.6 years and 49% male in controls vs. 41.0 ± 1.1 years and 56% male in the combined HCV/CFS group) (*Age*: *t* (165) = -0.88, *p* =  0.93; *Gender*: χ^2^ (1) = 0.79, *p* =  0.37). A full table of sample characteristics for the HCV cohort, and RF, PF, CFS and healthy control groups can be found in Supplementary Table 1.

### 2.3 Clinical questionnaires

The Chalder Fatigue Questionnaire (CFQ; Chalder et al., 1993) was used to measure the severity of fatigue in all groups, longitudinally (in the HCV group) and in the cross-sectional assessment. Scores of ≤18 are considered to be within the normal range for fatigue (Cella and Chalder, 2010). The Mini International Neuropsychiatric Interview (MINI) Major Depression section was administered to diagnose a previous history of depression or the occurrence of IFN-α-induced depression (Sheehan et al., 1998). The Brief Life Events (BLE) scale was administered to assess recent stressful events before starting IFN-α (Brugha and Cragg, 1990). The Childhood Experiences of Care and Abuse Questionnaire (CECA-Q) was used to collect information about childhood trauma, in all groups (Bifulco et al., 2005). The Inventory of Depressive Symptomatology (IDS) was used to measure depressive symptoms at treatment week 12 of IFN-α treatment in the HCV group (Rush et al., 1996). For sample characteristics (see Supplementary Table 1), the Socio-Demographic Schedule (SDS) was used to record age, gender, ethnicity and employment and relationships status (Mallett et al., 2002). Family history of mental illness was assessed using the Family Interview for Genetic Studies (Maxwell, 1992), and smoking and history of opioid use using a modified version of the Cannabis Experience Questionnaire (CEQ) (Barkus et al., 2006).

### 2.4 HCV measures

The HCV RNA viral load test obtained from medical records was used to assess illness severity at baseline, and treatment response. The viral load is the number of viral particles per ml of blood, presented in millions (AmpiliPrep, Roche), where a result of <15IU/ml equates to undetected. The test is used at treatment week 4 to assess Rapid Virological Response (undetected at week 4, indicative of likely later treatment success), and Sustained Virological Response (undetected six-months post-treatment, determines treatment outcome). We also examined the baseline Fibroscan result, an assessment of liver stiffness indicating the degree of liver damage.

### 2.5 Biological markers

In the HCV cohort, serum cytokine levels were measured at treatment week (TW)-0, TW4, TW24 and follow-up, and plasma kynurenine pathway metabolites at TW0, TW8, TW24 and at follow-up. In CFS patients and controls, all biological markers were measured once**,** at the cross-sectional assessment.

#### 2.5.1 Cytokines

Blood samples were collected using 6 ml BD vacutainer plastic tubes (silica clot activator) and left to clot for at least 30 min at room temperature. The blood was then centrifuged at 1850 *g* for 10 min at room temperature, and sera aliquots were stored at −80 °C for later analysis. Cytokines were measured using Meso Scale Discovery (MSD) V-PLEX sandwich immunoassays, and plates read on an MSD QuickPlex SQ 120. Samples were diluted two-fold, and measured in duplicate. MSD Pro-inflammatory Panel 1 (human) kits were used for the measurement of IFN-γ, IL-1β, IL-2, IL-4, IL-6, IL-8, IL-10, IL-12p70, IL-13, and TNF-α, and a custom Cytokine Panel 1 (human) kit was used for the measurement of IL-7, IL-17 A and VEGF. For the longitudinal study, all samples from the same patient were analysed together, though due to the volume of samples, samples were analysed in different batches, on different days; however, plates were always balanced for the presence of subjects from each group. The inter-assay coefficient of variations were <10%. Levels of IL-4 and IL-1β were not detected or below the lower limit of detection for most samples, and data are thus not reported here.

#### 2.5.2 Kynurenine pathway metabolites

Blood samples were collected using 9 ml VACUETTE® plasma separation, sodium heparin tubes. Samples were centrifuged at 500 *g* for 10 min at room temperature, and then plasma aliquots were stored at -80 for later analysis. The chromatographic system was composed of a Waters Acquity UPLC separations module connected to a Xevo TQ MS triple-quadrupole mass spectrometer, equipped with a Z-spray ESI ion source (Waters Corp., Milford, MA, U.S.). Separation was carried out using a Kinetex XB-C18, 2.6 μm 2.1 x 150 mm column (Phenomenex, Torrance, California, U.S.). Analysis of metabolites was conducted using liquid chromatography with tandem mass spectrometry (LC–MS/MS). The analytes and internal standards were detected using the multi reaction monitoring (MRM) technique.

System operation, data acquisition and data processing were controlled using MassLynx V4.1 software (Waters, Milford, USA). Baseline and treatment measures were completed in the same individuals in the same batch. For some individuals in the HCV group, baseline and treatment assessments were analysed at different times; however, variation across batches was low for the included measures, and measurements conducted later were considered separately in the cross-sectional analysis. Levels of tryptophan, kynurenic acid, quinaldic acid, 3-hydroxykynurenine (3-HK), xanthurenic acid, picolinic acid and quinolinic acid were successfully measured; levels of 5-hydroxy-l-tryptophan, 5-hydroxyindole-3-acetic acid, anthranilic acid and 3-hydroxyanthranilic acid (3-HAA) were all below or around the limit of quantification, and data are thus not reported here. Raw values for kynurenine levels in some patients were above the upper limit of quantification (>549); outliers greater than three times the interquartile range were excluded, resulting in the exclusion of two patients, but otherwise these values were included.

### 2.6 Data analysis

All data were analysed with IBM SPSS statistical software version 22. Data are presented as mean ± standard error of the mean (SEM). For the longitudinal study, differences between the PF and the RF groups were examined with a repeated measures analysis of variance, followed by comparisons using independent t-tests or paired t-tests, as appropriate, where an effect of IFN-α was shown. For the cross-sectional study, one-way ANOVAs were performed, followed by post-hoc Tukey’s Honest Significant Difference (HSD) tests, or Games-Howell tests where Welch’s ANOVA results are reported. When attempts to address the positive skew of some datasets through transformation were not successful, a Kruskal-Wallis or Mann-Whitney *U* test was performed, as appropriate. For all categorical variables, chi-squared (χ^2^) tests were used. Mixed-model analysis was used for confirmatory analyses when needed.

## 3 Results

### 3.1 Subjects who later develop PF have higher fatigue in response to IFN-α

**Fig. 1:**
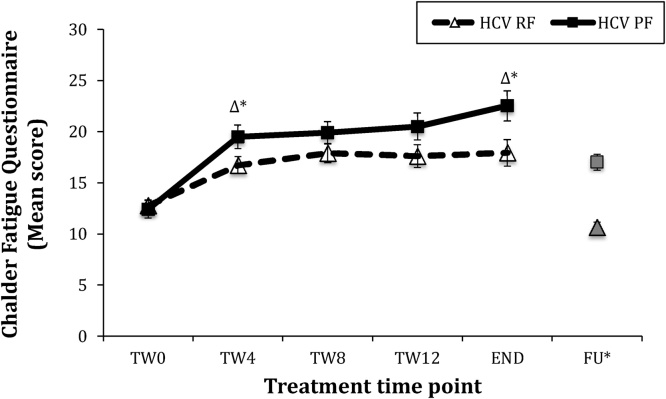
Fatigue scores in HCV Persistent Fatigue vs. Resolved Fatigue groups. **Notes-** error bars SEM; RF *n* = 29; PF *n* = 17; **HCV** – Hepatitis C Viral infection; Δ* - Δ vs. Treatment Week (TW)-0, *p* < 0.05; ‘END’ – composite end of treatment variable (see methods); **TW8 & FU** not included in ANOVA, included for illustrative purposes only.

We first analysed the levels of fatigue, before and during IFN-α, in subjects who later developed persistent fatigue (PF), and those who did not (resolved fatigue; RF) (see Fig. 1). At baseline (before IFN-α), levels of fatigue in the two groups were indistinguishable (*p* = 0.69). Moreover, there was the expected effect of time (*F* (2.58, 113.43) = 26.53, *p* < 0.001), confirming that IFN-α increased fatigue in both groups. However, the PF group did show an accelerated increase in fatigue levels by TW4. Specifically, there was a significant time X group interaction (*F* (2.58, 113.43) = 3.02, *p* = 0.040) driven by PF subjects experiencing a greater increase in symptoms over the first four weeks compared with RF patients (Δ TW0 vs. 4; PF: 7.1 ± 1.5 vs. RF: 4.0 ± 0.8, *p* = 0.046), with a statistical trend towards higher fatigue in the PF subjects at TW4 (19.5 ± 1.2 vs. 16.7 ± 0.8, *p* = 0.057). They also had a greater increase in fatigue by the end of treatment, relative to baseline (Δ TW0 vs. End = PF: 10.1 ± 1.8 vs. RF: 5.1 ± 1.1, *p* = 0.016), and fatigue scores were significantly higher in PF versus RF patients at the end of treatment (22.5 ± 1.5 vs. 17.9 ± 1.3, *p* = 0.035). Of note, PF was not due to worse recovery from the effects of IFN-α treatment per se, since the change in fatigue over the six-months post-treatment showed a similar rate of recovery in both groups (Δ END vs FU -7.3 ± 1.3 vs. -5.3 ± 1.4, *t* (49) = -0.96, *p* = 0.34).

Finally, we examined levels of depressive symptoms at week 4, at the same time as the increases in fatigue, and week 12, determined by previous research to be the time at which depressive symptoms induced by IFN-α reach peak levels. Levels at week 4 were no different between groups (RF vs. PF, mean ± SEM; 18.9 ± 2.0 vs. 19.9 ± 3.1, *t* (51) = -0.31, *p* = 0.76), and levels at week 12 were numerically higher in the PF group but with trend-statistical significance (20.09 ± 2.53 vs. 28.00 ± 17.51, *t* (49) = -1.73, *p* = 0.09). Taken together, this suggests that fatigue and depression are at least partially distinct

### 3.2 Subjects who later develop PF have higher IL-10 levels before treatment, and IL-6 and IL-10 levels in response to IFN-α

**Fig. 2:**
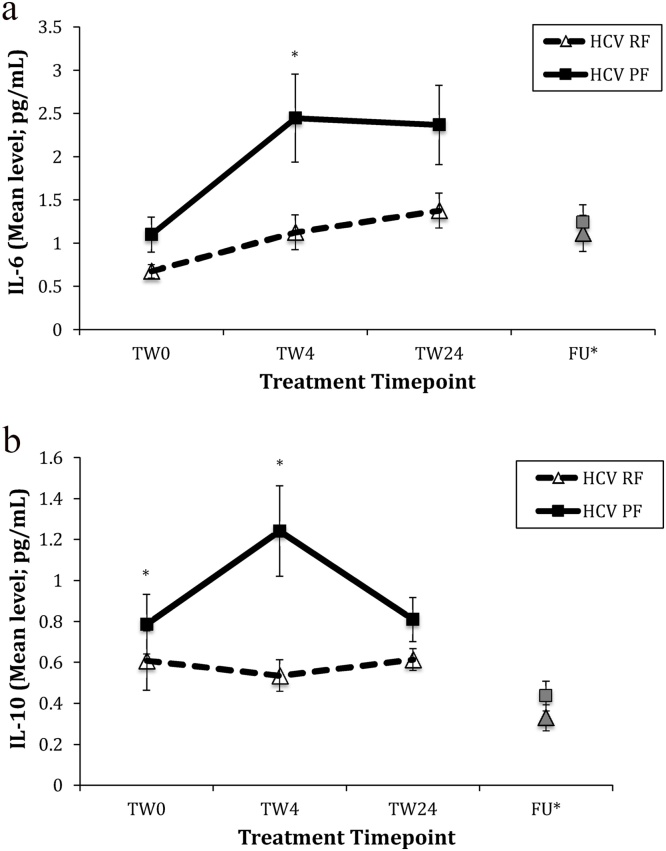
(a): Changes in IL-6 in HCV RF vs. PF groups. (RF *n* = 20; PF *n* = 12) and (b) Changes in IL-10 in HCV RF vs. PF groups. (RF *n* = 18; PF *n* = 12). * Mann Whitney *U* test *p* < 0.05; FU* - not included in ANOVA, included for illustrative purposes only; **HCV** – Hepatitis C Viral infection, **TW** – Treatment Week, **IL** – Interleukin.

At baseline, there was a statistical trend towards higher IL-6 levels in the PF group, as well as statistically higher IL-10 levels, compared with RF patients (see Fig. 2a and b; IL-6, *p* = 0.073; IL-10, *p* = 0.034). There were no other baseline differences in the other cytokines measured (see Table 1).

**Table 1:** Baseline comparison of cytokine levels in HCV Resolved fatigue vs. Persistent fatigue groups.

Biological Measure	HCV RF	HCV PF	Test and statistic
	Mean ± SEM		
IFN-γ	7.85 ± 1.45	9.68 ± 2.67	*U* = 66, *z* = -0.77, *p* = 0.44
IL-2	0.22 ± 0.05	0.24 ± 0.07	*U* = 110, *z* = -0.39, *p* = 0.70
IL-6	0.68 ± 0.08	1.1 ± 0.2	*U*= 74, *z*= -1.79, *p*= 0.073
IL-7	16.65 ± 2.34	14.58 ± 1.89	*U* = 153, *z* = -0.03, *p* = 0.97
IL-8*^^^*	13.56 ± 1.88	15.09 ± 2.31	*U* = 108, *z* = -1.01, *p* = 0.31
IL-10	0.61 ± 0.14	0.79 ± 0.15	***U* = 58, *z* = -2.12, *p* = 0.034**
IL-12p70	0.09 ± 0.02	0.23 ± 0.07	*U* = 32, *z* = -1.34, *p* = 0.18
IL-13	0.33 ± 0.08	0.23 ± 0.08	*U* = 94, *z* = -1.15, *p* = 0.25
IL-17A	1.82 ± 0.51	1.61 ± 0.32	*U* = 91, *z* = -1.31, *p* = 0.19
TNF-α	4.17 ± 0.43	4.9 ± 0.65	*U* = 108, *z* = -1.01, *p* = 0.31
VEGF	200.19 ± 36.31	217.17 ± 52.81	*U* = 94, *z* = -0.05, *p* = 0.96

Notes - bold denotes significant test result *p* > 0.05; underlined denotes statistical trend; HCV – Hepatitis C Viral infection; IFN – Interferon; IL – Interleukin; TNF – Tumour Necrosis Factor; VEGF – Vascular Endothelial Growth Factor.

For IL-6, there was a significant effect of time (*F* (2, 60) = 12.53, *p* < 0.001), indicating an increase in response to IFN-α in all subjects. There was no significant interaction between time and group on levels of IL-6 (*F* (2, 60) = 2.17, *p* = 0.12). However, there was a significant effect of group (*F* (1, 30) = 9.73, *p* = 0.004), indicating that levels were different between PF and RF. Post-hoc tests revealed that IL-6 values in PF subjects increased to double those of RF patients by TW4 (*p* = 0.011) and remained higher at TW24 (*p* = 0.094) (see Fig. 2a). To confirm these observations, we supplemented this ANOVA analysis (on the sample for which IL-6 measures were available at all time-points) with the assessment of a fixed effect interaction in a mixed model, in the wider group for which IL-6 measurements were available for at least one time-point (RF = 34; PF = 15). Employing an autoregressive covariance structure to account for the repeated measures within patients, we did find a significant interaction between time and group *(F* (5,77) = 5.57, *p* <0.001), thus confirming the notion of a differential effect of IFN-α on IL-6 levels based on ‘persistent fatigue’ status.

For IL-10, there was no effect of time (*F* (1.58, 44.20) = 1.65, *p* = 0.21), and there was a trend towards an interaction between time and group (*F* (1.58, 44.20) = 3.23, *p* = 0.057). However, there was a significant effect of group (*F* (1, 28) = 8.86, *p* = 0.006), indicating again that levels were different between PF and RF patients. As for IL-6, levels in PF patients increased to more than double those of RF patients by TW4, (*p* = 0.001), though in contrast were reduced to similar levels across groups by TW24 (*p* = 0.138) (see Fig. 2b). Using the same approach described above to confirm these observations model in the wider group for which IL-10 measurements were available for at least one time-point, the mixed model demonstrated a slightly more significant interaction effect, although still at a statistical trend level (*F* (5, 82) = 2.29, *p* = 0.053).

Of note, these early, greater increases in both cytokines in those who later developed PF occur concurrently to the greater increases in fatigue over the same period. Spearman’s rho correlation analyses including the delta scores (Δ 0 vs. 4) representing change in fatigue and (i) IL-6 and (ii) IL-10 were performed to explore this link further. In the whole group, there was no association between changes in fatigue and IL-6 (*r_s_*= -0.13, *p* = 0.41), nor with changes in IL-10 (*r_s_* = 0.13, *p* = 0.39). With regards to the effect of IFN-α on other cytokines, only levels of IL-2, IL-8, IL-17 A and TNF − α increased in response to IFN-α, as shown by significant effects of time (*p* < 0.005 for all comparisons, see Supplementary Table 3). However, there were no interactions between time and group, nor was there an effect of group, indicating similar changes in both PF and RF groups during IFN-α. There was no effect of IFN-α on other cytokines (see Supplementary Table 3).

### 3.3 In the cross-sectional study, patients with the established PF phenotype and patients with CFS do not show any increase in cytokine levels compared with control conditions

Having shown that patients who later go on to develop PF have higher IL-10 before treatment, and higher levels of IL-6 and IL-10 during IFN-α treatment, compared with RF patients, we wanted to test whether these cytokines continue to be higher in the PF group at follow-up (six-months post-cessation of IFN-α), when the PF phenotype is established; moreover, we wanted to compare levels of cytokines also with those in patients with CFS and in controls.

In terms of fatigue severity (measured with the CFQ), there were significant differences between groups (ANOVA, *F* (3, 58.4) = 154.4, *p* < 0.001), with CFS patients having the most severe symptoms (26.0 ± 0.6; *p* < 0.001 vs. all other groups) and the PF patients with an intermediate CFS-like phenotype (17.0±0.8; *p* < 0.001 vs. RF and controls), while RF and controls were, expectedly, the lowest and indistinguishable (10.6±0.5 and 11.6±0.3, respectively). The more severe fatigue in CFS patients than in the PF patients is understandable, considering that they had been referred to a specialist service for treatment, and the longer length of illness in this group (84±12 months in CFS vs. 6 months in the PF group).

Table 2 presents cytokine levels in the four groups. To limit the number of statistical comparisons, only the cytokines that were significantly regulated by IFN-α were analysed in the cross-sectional assessment.

**Table 2:** Cross sectional comparison of cytokine levels.

Measure	CTRL	HCV RF	HCV PF	CFS	Kruskal-Wallis *Post-hoc test (MWU)*
	Mean ± SEM				
IL-2	0.24 ± 0.02	0.33 ± 0.07	0.37 ± 0.11	0.20 ± 0.02	χ^2^ (3) = 5.39, *p* = 0.15
IL-6	0.53 ± 0.03	1.21 ± 0.22	1.40 ± 0.21	0.44 ± 0.03	**χ^2^ (3) = 32.62,** ***p*<0.001** CFS < PF, RF*** CTRL < PF***, RF*
IL-7	20.98 ± 0.94	15.77 ± 2.00	16.59 ± 2.87	15.85 ± 0.87	**χ^2^ (3) = 20.05,** ***p*<0.001** CTRL > PF*, RF, CFS***
IL-8	9.74 ± 0.38	13.64 ± 1.73	14.94 ± 2.55	8.47 ± 0.39	**χ^2^ (3) = 10.23,** ***p* = 0.017** CFS < PF, RF, CTRL*
IL-10	0.29 ± 0.01	0.52 ± 0.09	0.57 ± 0.12	0.27 ± 0.02	**χ^2^ (3) = 15.14,** ***p* = 0.002** CFS < PF***; RF* CTRL < PF***
IL-17A	0.81 ± 0.04	1.89 ± 0.37	2.04 ± 0.45	0.88 ± 0.06	**χ^2^ (3) = 20.81,** ***p*<0.001** CFS < PF, RF** CTRL < PF, RF***
TNF-α	2.54 ± 0.07	4.57 ± 0.49	5.09 ± 0.71	2.61 ± 0.09	**χ^2^ (3) = 40.91,** ***p*<0.001** CFS < PF, RF*** CTRL < PF. RF***

**Notes -** cytokines not regulated by IFN-α not measured in CS study; **bold** denotes significant test result *p* < 0.05; post-hoc comparisons * *p* < 0.05; ** < 0.01; *** <0.001; CTRL – healthy control; HCV – Hepatitis C Viral infection; RF – Resolved Fatigue; PF – Persistent Fatigue; CFS – Chronic Fatigue Syndrome; IL – Interleukin; TNF – Tumour Necrosis Factor.

With regards to IL-10 and IL-6 (the cytokines that distinguished PF from RF), there were significant differences in the levels of IL-6 and IL-10 between the four groups (ANOVA, *p* < 0.001 and *p* = 0.002, respectively), but PF patients were *not different* from RF patients, even if the CFS-like phenotype was established at this time. Moreover, CFS patients had levels that were lower (IL-6) or indistinguishable (IL-10) from healthy volunteers (see Table 2). The significant differences between the groups were driven by levels of IL-6 and IL-10 being higher in *both* PF and RF compared with both CFS and controls (see Table 2).

With regards to levels of IL-2, IL-8, IL-17 A and TNF-α (the cytokines that were regulated by IFN-α but did not distinguish between PF and RF), for IL-2 there were no significant differences between groups (*p* =  0.14), while for the others there were significant differences between the groups (*p* values ranging <0.001 - 0.003, see Table 2), and differences were all similar to those described for IL-6 and IL-10, i.e., higher in both PF and RF groups compared with CFS and controls, but *not different* between PF and RF or between CFS and controls.

Taken together, these findings support the notion that the presence of the CFS phenotype *per se* does not lead to increased inflammation at the time in which the CFS phenotype is present, for both IFN-α-induced persistent fatigue and clinically-defined CFS. Interestingly, the higher levels of five out of six cytokines in both PF and RF subjects indicate a persistent immune activation six months post-cessation of IFN-α, which is unrelated to fatigue. Of note, this persistent immune activation is likely due to IFN-α, considering the absence of HCV at this time-point in the majority of cases *(see point 5)*.

### 3.4 Kynurenine pathway metabolites change with IFN-α treatment but do not distinguish PF from RF or CFS from controls

Of the kynurenine pathway markers examined during IFN-α treatment, there were no baseline differences between the RF and PF groups (data not shown; levels reported in Supplementary Table 4).

During treatment, there was an effect of time (indicating regulation by IFN-α) on several markers. There was an increase over time in the levels of kynurenine and of 3-Hydroxykynurenine (3-HK), and of the ratio of kynurenine to tryptophan (KYN/TRP ratio), and a decrease over time in kynurenic acid and quinaldic acid (*p* values ranging <0.001 – 0.03). There were no significant interactions or group effects, indicating that, where levels did change, there was a similar effect of IFN-α treatment in both PF and RF subjects (see Supplementary Table 4).

In the cross-sectional analysis, again to limit the number of statistical comparisons we only analysed the metabolites that were significantly regulated by IFN-α (see Supplementary Table 4). Notwithstanding this conservative approach, this is, to the best of our knowledge, the largest characterisation of plasma kynurenine metabolites in CFS patients. There were significant differences between the groups for kynurenine, tryptophan, KYN/TRP ratio, 3-HK, and kynurenic acid (*p* values ranging <0.001 – 0.004), but not for quinaldic acid (*p* =  0.99). As for the cytokines described above, levels tended to be higher in PF and RF compared with the other groups, but indistinguishable between PF and RF. Interestingly this was true even of kynurenic acid and quinaldic acid, which had lowered during IFN-α treatment, but were either higher or at a similar level to baseline (respectively) when measured again six-months post-treatment.

CFS patients tended to have values that were lower (KYN/TRP ratio and 3-HK) or indistinguishable (tryptophan, kynurenic acid) than controls.

Taken together, these analyses confirm the activation of the kynurenine pathway in response to an immune trigger, but do not support a role in CFS and CFS-like syndromes.

### 3.5 Psychosocial and clinical risk factors do not distinguish PF from RF subjects

Having provided evidence that PF patients have higher levels of IL-10 at baseline, and go on to develop more severe fatigue (and stronger increases in IL-6 and IL-10) already by TW4, we proceeded to investigate which psychosocial or clinical factors, if any, could explain the increased sensitivity of PF patients to IFN-α. The psychosocial and clinical characteristics of the PF and RF groups were similar across all variables examined (data not all shown; see also Supplementary Table 1 for descriptive data).

Of note, there was no difference in the rate of personal history of depression (PF 44% vs. RF 32%, *p* = 0.39) or in the proportion of patients who reported having experienced at least one stressful life event in the six-months before the initiation of treatment (PF 39% vs. RF 49%, *p* = 0.50). Nor was there a difference in the proportion of patients who reported at least one form of early life trauma (loss of parent, separation from parent, physical and/or sexual abuse) (PF 50% vs. RF 49%, *p* = 0.93).

In terms of HCV-related clinical factors, there were no significant differences between groups in any virus or treatment characteristics, HCV illness severity, or the measures of HCV treatment response to IFN-α (data not shown; see Supplementary Table 2 for summary information). Most notably, there was no relationship between IFN-α induced PF and treatment outcome (virus undetectable in the blood six-months post-treatment; PF 94% vs. RF 84%, *p* = 0.27). Incidentally, there was also no difference in the rate of IFN-α induced depression (PF 50% vs. RF 33.3%, *p* = 0.24), suggesting a distinction between this and the PF phenotype.

## 4 Discussion

This is the first study to investigate IFN-α induced *persistent* fatigue as a proxy model of Chronic Fatigue Syndrome. We find that around 30% of patients reported persistent fatigue (PF) at the follow-up six-months after the end of IFN-α treatment, despite the original immune stimulus no longer being present. We also find that those who developed PF have a more rapid increase in fatigue early during IFN-α treatment, as well as higher IL-10 levels before treatment, and IL-10 and IL-6 levels early on during IFN-α treatment – that is, more than one year before the PF phenotype is established. However, this initial immune activation is no longer associated with PF at the six-month follow-up, when the PF phenotype is established, nor is it present in a group of CFS patients used as a clinical reference group.

Of note, PF is not due to worse recovery from the effects of IFN-α treatment per se, since the changes in clinical symptoms over the six-months post-treatment reveal a similar rate of recovery in both the PF and the RF groups. Our study confirms and extends findings from previous longitudinal studies of post-infective fatigue syndrome, which have observed greater fatigue at initial presentation to clinics, around four weeks post-infection, in those who would go on to experience persistent fatigue (Candy et al., 2003; Hickie et al., 2006; Vollmer-Conna et al., 2007). Interestingly, by adding a ‘baseline’ assessment before IFN-α (i.e., equivalent to a hitherto unavailable fatigue measurement before the infection), our study shows that pre-treatment levels of fatigue are *not* a good predictor of the development of CFS in response to an immune challenge.

We also find increased (serum) IL-10 in PF patients prior to IFN-α treatment, and an exaggerated response in IL-10 and IL-6 to IFN-α during treatment, with higher levels driven by a greater increase in the first four weeks. The higher TW4 levels of IL-6 in PF persist at TW24, and represent a greater response in the general IFN-α-induced increase in IL-6 that is present in all patients. In contrast, the higher TW4 levels of IL-10 in PF subside by TW24 and are only present in the PF group, suggesting that the IFN-α-induced increase in this cytokine indicates a specific sensitivity to an immune stimulus in those who are also at risk of fatigue-like syndromes. It is also of note that we have recently published, in a different but overlapping sample of patients taking IFN-α, that the IFN-α-induced increase in IL-6 levels is *not* related to the development of IFN-α-induced depression (Hepgul et al., 2016a, 2016b). Thus, this indicates a specific role of IL-6 in the development of fatigue. Indeed, also in light of our finding that PF was not associated with IFN-α-induced depression in the present study, these cytokine data provide further evidence for a clear distinction between these two phenotypes. As previously extensively discussed (Dantzer et al., 2014), IL-6 has been noted as being particularly relevant to fatigue, in relation to IFN-α induced symptoms as well as other clinical contexts including cancer. We have also recently shown that IL-6 activates a number of downstream molecular targets, such as signal transducer and activator of transcription-1 (STAT1) and aquaporin 4 (Borsini et al., 2018), and these should be investigated in the context of CFS.

Interestingly, previous studies of post-infective fatigue syndrome from the Dubbo Infectious Outcomes Study (DIOS) cohort found no association between acute fatigue and peripheral cytokines in serum, nor did they find differential levels of cytokines in patients with established post-infective fatigue versus those in whom fatigue resolved (Vollmer-Conna et al., 2007, 2004). The increased cytokine levels at the time of acute fatigue in our sample, but not in the DIOS study, may be attributable to the greater increases in cytokines in response to therapeutic doses of IFN-α versus the natural immune response to infection (Pollmacher et al., 2002). However, the lack of differences in cytokine levels between patients with established post-infective fatigue and those in whom fatigue resolved in the DIOS study is consistent with our study, as we also find that cytokines levels do not distinguish PF from RF patients at the 6-month follow-up (nor CFS patients from controls). Taken together, these lines of evidence confirm the notion that, in CFS, increased immune activation at the time of onset is more relevant than at the time of the established phenotype. This could explain the inconsistent findings in the search for an immune abnormality once CFS is established. To date, and after a multitude of studies, only TGF-β has been consistently found raised in CFS (Blundell et al., 2015; Montoya et al., 2017).

Our finding that levels of IL-10 were already higher at baseline in the PF group, and that baseline levels of IL-6 may also be raised, in the absence of higher baseline fatigue, may be explained in part by a genetic predisposition. While it does not cause fatigue in itself, greater fatigue is induced in response to a strong immune challenge, which then persists. Indeed, this has been explored by studies examining the variability in response to the same immune challenge. Genetic polymorphisms in IL-6 (IL-6 −174 G allele, rs1800795) and IL-10 genes (-592C allele, rs1800872) have been associated with higher levels of these markers (Barnes et al., 2017). A study found a protective effect of the ‘low IL-6’ synthesizing genotype (CC) of a polymorphism in the promoter region of the IL-6 gene (rs1800795), on IFN-α induced depressive symptoms, although in this case, there was no association with IFN-α induced acute fatigue (Bull et al., 2009). Another study found no association between rs1800795 and CFS (Carlo-Stella et al., 2006) or acute fatigue in a PIFS cohort (Piraino et al., 2012), though it was linked with greater mood disturbance during the sickness phase (Piraino et al., 2012). A study of the Dubbo Infectious Outcomes Study (DIOS) post-infective fatigue syndrome (PIFS) cohort found that the interleukin-10 -592C/A polymorphisms were significantly associated with illness severity, cytokine protein levels and the duration of illness in those with an infectious disease. However, somewhat converse to our findings, the authors observed that those with the genotype associated with higher production of IL-10 (-592CC) were at lower risk of experiencing severe symptoms during the acute illness phase, versus those with the CA or AA genotypes (Vollmer-Conna et al., 2008). A study of a cohort from the same group found that individuals with the -592C allele were also less likely to experience neurocognitive effects or mood disturbances during the acute sickness phase (Piraino et al., 2012). Of note, however, is that in our PF group, while IL-10 did only appear to increase in PF patients, this effect was only in the initial response, and did decline over time, perhaps indicative of the effects of different cytokines and different points in response to IFN-α.

This study also presents, to our knowledge, one of the largest characterisations of plasma kynurenine metabolites in patients with CFS or a CFS-like syndrome. With reference to longitudinal changes in response to IFN-α, while levels of some of the kynurenine pathway metabolites changed (KYN/TRP ratio, kynurenic acid, quinaldic acid and 3-HK), confirming the well-established activation of the kynurenine pathway, these changes were not associated with the later development of PF. Interestingly, previous studies have shown that activation of the kynurenine pathway *is* associated with depression and somatic symptoms, including fatigue, *during* IFN-α (Anderson et al., 2014; Leonard and Maes, 2012) or viral infection with Chronic Active Epstein Barr virus (Bellmann-Weiler et al., 2008). Indeed, the latter study found that lower tryptophan and higher indoleamine 2,3-dioxygenase (IDO)-activity were associated with more severe symptoms (tiredness, fever and night sweats) *during* the Chronic Epstein Barr infection. However, likely due to low numbers experiencing post-infective fatigue (4–8 months later), they did not assess any longitudinal relationship between early IDO activation and later post-infective fatigue (Bellmann-Weiler et al., 2008).

In our cross-sectional comparison, again we find a similar pattern as seen for cytokines, that is there is no increased activation of the kynurenine pathway in PF (compared with RF) or CFS patients (compared with controls), although both PF and RF showed evidence of activation (compared with the other two groups). Interestingly, however, levels of the KYN/TRP ratio and 3-HK were *lower* in CFS patients than controls, while the tryptophan levels were similar to controls. This lower KYN/TRP ratio in CFS (and the lack of association with PF) is somewhat in opposition to a study of somatization which observed higher levels of the KYN/TRP ratio (Maes et al., 2011). This evidence points again to a different biological underpinning of primarily psychiatric syndromes, such as depression and somatization, as opposed to CFS. This notion is further supported by the lack of an association, in our study, between PF and any of the classic, stress-related risk factors for psychiatric disorders. Targeting the kynurenine pathway, through antidepressants or nutritional interventions, may thus not be a relevant therapeutic strategy in CFS (Borsini et al., 2017).

The prospective design gives this study a unique ability to measure fatigue changes in individuals while also taking into consideration baseline (pre-morbid) symptoms, and it is further strengthened by the exclusion of patients with other conditions that can impact fatigue or inflammation, and the presence of the clinical reference group of patients with CFS. Nevertheless, a few limitations should also be mentioned. First, we acknowledge that we can only speculate at this stage on whether or not the mechanisms underlying the persistence of fatigue in CFS and IFN-α induced PF are related. However, in order to progress in the field of CFS/ME it is vital to take advantage of opportunities to learn more from other overlapping conditions, and it is especially important to collect evidence that could be relevant for earlier stages of CFS. The benefit of our proxy model is this opportunity to capture measures before and during the response to an early immune trigger, which would be impossible to achieve in a CFS population within the remit of available resources. Nevertheless, this remains a ‘proxy model’ and its validity and relevance for patients with CFS must be confirmed by converging evidence from other, similar proxy-model studies. In addition, the differences between the HCV and CFS populations were such that it was not possible to recruit a matched CFS group (e.g. 80 vs. 31% males between groups). The healthy control group were therefore matched to the combined HCV and CFS group. Also of relevance, the CFS sample was recruited from a specialist service, and thus had an established diagnosis and long (average of 7 years) duration of illness, and as such were different from the PF groups that only had 6 months of post- IFN-α fatigue. However, in using CFS patients recruited in this way, we could ensure that all patients were diagnosed in a standardised manner, by trained doctors who were experienced in conducting such assessments. Of note, our approach to categorise fatigue as ‘resolved’ (returned to the same level or lower than baseline), and ‘persistent’ (higher than baseline) may also attract some criticism given that this could mean that even a 1-point change on the CFQ can determine the classification. However, we believe this to be justified in order for us to focus on *change* in the individual in response to the trigger, where baseline fatigue is acknowledged, and in the context of additional changes in fatigue during IFN-α. It makes the best of the availability of baseline measurements in this smaller sample. The fact that we were able to identify group differences in cytokine changes during IFN-α treatment further strengthens the validity of our approach. Regarding the changes in IL-6 and IL-10, although the univariate analyses indicated a greater change in patients who would go on to experience persistent fatigue, these were not of significant magnitude to reach statistical significance in a ANOVA group x time interaction in the sample for which all cytokine measurements were available for all time-points. While a mixed model analysis in the larger samples for which cytokines measurements were available for at least one time-point showed a significant interaction for IL-6 and a stronger trend-level (*p* = 0.053) for IL-10, the lack of significant interactions in the primary analyses is a limitation that should be taken into consideration, and the finding should be replicated in a larger study. Finally, due to the sample size there was no correction for multiple comparisons; however, we aimed to limit the number of statistical comparisons by pre-selecting the cytokines to measure at the different stages of the study. One important point, though not necessarily a limitation as such, is that since the study was conducted there have been significant advances in the field of HCV treatment such that IFN-α free regimens now dominate, at least in developing countries. The direct acting antivirals (DAAs) referenced in our patient sample (Boceprevir, Telaprevir and Simeprevir) have since been overtaken by other more efficient, better tolerated DAAs. However, there are still cost considerations related to the prescription of the newer drugs. IFN-α and ribavirin remains an effective, cost effective option, and is still recommended for the treatment of mild chronic HCV, meaning that while more difficult, it may still be possible to replicate our findings in a similar cohort (National Institute for Health and Clinical Excellence, 2018). In addition, immune challenges such as typhoid vaccine and lipopolysaccharides can also be used in experimental medicine studies in order to replicate our findings.

In conclusion, findings from this study support the hypothesis that abnormal immune mechanisms are important in CFS, but only early in the course of the illness, around the time of the trigger, rather than when the syndrome is established. Moreover, our study confirms the importance of the acute fatigue response to the trigger, rather than of the recovery period preceding the illness. Future research will need to examine the molecular mechanisms that underlie an exaggerated immune response and that are involved in the conversion from acute to persistent fatigue symptoms.

## Conflict of interest

Prof. Foster has received speaker and consultancy fees from companies that market drugs to treat hepatitis C, specifically AbbVie, Gilead, Merck and Roche.

Dr Forton has received speaker consultancy fees from companies that market drugs to treat hepatitis C, including AbbVie, Gilead, BMS and Janssen. He has received funding for trials from Merck.

Dr Kosh Agarwal has received fees from companies that market drugs to treat Hepatitis C, including AbbVie, Gilead, Astellas, Intercept, Janssen, Merck and Achillon, and has received grants from AbbVie, Gileas, BMS and Roche.

Prof. Cleare has in the last three years received honoraria for speaking from Astra Zeneca and Lundbeck, honoraria for consulting from Allergan, Livanova, Lundbeck and Janssen, and research grant support from Lundbeck, the Medical Research Council (UK), Wellcome Trust (UK) and the National Institute for Health Research (UK).

Dr Harrison has grant funding from Wellcome (NIMA), the Medical Research Council (UK), Arthritis Research UK and Action for ME. He has also acted as a consultant for GSK and has received research funding from Janssen.

Prof. Pariante and Dr. Zunszain have received research funding from Johnson & Johnson as part of a program of research on depression and inflammation, and research funding from the Medical Research Council (UK) and the Wellcome Trust for research on depression and inflammation as part of two large consortia that also include Johnson & Johnson, GSK and Lundbeck.

There are no other conflicts of interest.
