# Supplementary material for: Persistent fatigue induced by interferon-alpha: a novel, inflammation-based, proxy model of chronic fatigue syndrome
Source: Psychoneuroendocrinology. 2019 Feb;100:276–85. doi: 10.1016/j.psyneuen.2018.11.032 (PMC6350004; doi:10.1016/j.psyneuen.2018.11.032)
Supplement: Supplementary file 1 [file mmc1.docx]

***Supplementary table 1: socio-demographic characteristics***

| ***Characteristic*** | **HCV (all)**  *n* = 55 | ***Subgroups*** | | **CFS**  *n* = 54 | **CTRL**  *n* = 57 |
| --- | --- | --- | --- | --- | --- |
|  |  | **HCV RF**  *n* ­= 37 | **HCV PF**  *n* ­= 18 |  |  |
| **Age (years)**  Mean±SEM | 44.6±1.60 | 43.7±2.0 | 46.4±2.8 | 37.2±1.5 | 40.8±1.6 |
| **Gender**  Male | 44 (80%) | 28 (75.7%) | 16 (88.9%) | 17 (31.5%) | 28 (49.1%) |
| **Ethnicity**  White British/Irish | 28 (50.9%) | 16 (43.2%) | 12 (66.7%) | 39 (72.2%) | 32 (56.1%) |
| **Education** **level**  Unemployed | 19 (34.5%) | 12 (32.4%) | 7 (38.9%) | 20 (37.7%) | 9 (15.8%) |
| **Relationship** **status**  Married/living with someone | 22 (40%) | 15 (40.5%) | 7 (38.9%) | 18 (34.0%) | 22 (38.6%) |
| **History** **of** **depression**  y/n | 20 (36.4%) | 12 (32.4%) | 8 (44.4%) | 33 (62.3%) | 24 (42.1%) |
| **Depressive symptoms**  Mean±SEM | 12.3±1.5 | 8.9±1.4 | 19.4±2.9 | 28.3±1.7 | 7.5±0.7 |
| **Family** **history** **of** **mental** **illness**  First degree | 17 (30.9%) | 11 (33.3%) | 6 (35.3%) | 36 (70.6%) | 31 (54.4%) |
| **History of opioid abuse**  y/n | 24 (43.6%) | 15 (41.7%) | 9 (56.3%) | 1 (1.9%) | 1 (1.8%) |
| **Current smoker**  y/n | 22 (40%) | 13 (36.1%) | 9 (52.9%) | 10 (18.9%) | 10 (17.5%) |

***Notes -*** *HCV – Hepatitis C Viral infection; PF – Persistent Fatigue; RF – Resolved Fatigue; CFS – Chronic Fatigue Syndrome; CTRL – healthy control*

***Supplementary table 2: clinical characteristics in the HCV sample***

|  | **HCV (all)**  *n* = 55 | ***Subgroups*** | |
| --- | --- | --- | --- |
|  |  | **HCV RF**  *n* ­= 37 | **HCV PF**  *n* ­= 18 |
|  |  |  |  |
| **HCV genotype** |  |  |  |
| 1 | 10 (18.2%) | 6 (16.2%) | 4 (22.2%) |
| 2 | 9 (16.4%) | 4 (10.8%) | 5 (27.8%) |
| 3 | 35 (63.6%) | 26 (70.3%) | 9 (50.0%) |
| 4 | 1 (1.8%) | 1 (2.7%) | 0 (0.0%) |
|  |  |  |  |
| **HCV viral load, millions** |  |  |  |
| Mean±SEM | 2.8±0.5 | 2.7±0.6 | 3.0±0.8 |
|  |  |  |  |
| **Liver stiffness, kPa** |  |  |  |
| Mean±SEM | 8.5±0.9 | 8.6±1.1 | 8.4±1.3 |
|  |  |  |  |
| **Treatment type** |  |  |  |
| IFN-α + ribavirin | 47 (85.5%) | 31 (83.8%) | 16 (88.9%) |
|  |  |  |  |
| Triple (as above, plus DAA) | 8 (14.5%) | 6 (16.2%) | 2 (11.1%) |
| *Telaprevir* | 5 | 4 | 1 |
| *Simeprevir* | 2 | 1 | 1 |
| *Boceprevir* | 1 | 1 | 0 |
|  |  |  |  |
| **Treatment outcome** |  |  |  |
| Rapid Virological Response | 33 (60%) | 24 (64.9%) | 9 (50.0%) |
|  |  |  |  |
| Sustained Virological Response | 48 (87.3%) | 31 (83.8%) | 17 (94.4%) |
|  |  |  |  |

***Notes -*** *HCV – Hepatitis C Viral infection; PF – Persistent Fatigue; RF – Resolved Fatigue; IFN-α – Interferon-alpha; DAA - Direct Acting Anti-viral; Rapid Virological Response – HCV undetectable at Treatment Week 4, indicator of expected success of treatment; Sustained Virological Response – HCV undetectable six-months post-treatment, determines outcome of treatment*

***Supplementary Table 3: cytokine assay detection limits***

| MSD Cytokine measurement | Lower limit of detection (LLOD) |
| --- | --- |
|  | pg/mL |
|  |  |
| *IFN-γ* | 0.20 |
| *IL-1β** | 0.04 |
| *IL-2* | 0.09 |
| *IL-4** | 0.02 |
| *IL-6* | 0.06 |
| *IL-7* | 0.16 |
| *IL-8* | 0.04 |
| *IL-10* | 0.03 |
| *IL-12p70* | 0.11 |
| *IL-13* | 0.24 |
| *IL-17A* | 0.93 |
| *TNF-α* | 0.04 |
| *VEGF* | 1.12 |
|  |  |
| HPLC-MS/MS Kyn Pathway | Lower limits of quantification (LLOQ) |
|  | ng/mL |
|  |  |
| *3-HK* | 1 |
| *Kyn* | 100 |
| *Kynurenic acid* | 1 |
| *Pic acid* | 10 |
| *Quinaldic acid* | 1 |
| *Quinolinic acid* | 10 |
| *Tryptophan* | 8500 |
| *Xanthurenic acid* | 1 |
|  |  |

***Notes*** *- IFN – Interferon; IL – Interleukin; TNF – Tumour Necrosis Factor; VEGF – Vascular Endothelial Growth Factor; 3-HK – 3-Hydroxykynurenine; Kyn – Kynurenine; Pic – picolinic (acid); * insufficient detectable values for analysis, see methods; see product information on www.mesoscale.com*

***Supplementary Table 4: Changes in cytokines during IFN-α treatment in Persistent Fatigue vs Resolved Fatigue patients (repeated measures GLM)***

| Marker | HCV RF | | | HCV PF | | | Statistics | | |
| --- | --- | --- | --- | --- | --- | --- | --- | --- | --- |
|  | TW0 | TW4 | TW24 | TW0 | TW4 | TW24 | Time | Time X Group | Group |
| *IFN-γ* | 7.85±1.45 | 8.19±1.91 | 8.10±1.46 | 9.68±2.67 | 7.39±0.84 | 9.20±1.08 | *F* (2,50) = 0.55  *p* = 0.58  partial η^2^  = 0.02 | *F* (2,50) = 0.88  *p* = 0.42  partial η2 = 0.02 | *F* (1,25) = 0.09  *p* = 0.76  partial η^2^  = 0.004 |
| *IL-2* | 0.22±0.05 | 0.25±0.05 | 0.35±0.07 | 0.24±0.07 | 0.34±0.07 | 0.41±0.09 | ***F* (1.39, 41.54)^*^ = 17.33**  ***p<*0.001**  partial η^2^  = 0.37 | *F* (1.39, 41.54)^*^ = 1.21  *p*=0.30  partial η^2^  = 0.04 | *F* (1,30) = 0.38  *p* = 0.54  partial η^2^  = 0.01 |
| *IL-6* | 0.68±0.08 | 1.13±0.20 | 1.34±0.20 | 1.10±0.20 | 2.45±0.51 | 2.37±0.46 | ***F* (2, 60) = 12.53**  ***p*<0.001**  partial η^2^  = 0.30 | *F* (2, 60) = 2.17  *p* = 0.12  partial η^2^  = 0.07 | ***F* (1,30) = 9.73**  ***p* = 0.004**  partial η^2^  = 0.25 |
| *IL-7* | 16.65±2.34 | 17.85±2.72 | 18.62±2.41 | 14.58±1.89 | 17.03±3.06 | 17.25±3.05 | *F* (2, 68) = 2.59  *p* =0.083  partial η2 = 0.07 | *F* (2, 68) = 0.17  *p* =0.84  partial η^2^  = 0.005 | *F* (1,34) = 0.16  *p* = 0.70  partial η^2^  = 0.005 |
| *IL-8* | 13.56±1.88 | 19.67±2.48 | 18.06±2.21 | 15.09±2.31 | 23.98±4.10 | 21.91±5.03 | ***F* (1.55, 49.68)^**^ = 7.31**  ***p* = 0.003**  partial η^2^  = 0.19 | *F* (1.55, 49.68)^•^= 3.23  *p* = 0.71  partial η^2^  = 0.008 | *F* (1,32) = 0.87  *p* = 0.36  partial η^2^  = 0.03 |
| *IL-10* | 0.61±0.14 | 0.54±0.08 | 0.61±0.05 | 0.79±0.15 | 1.24±0.22 | 0.81±0.11 | *F* (1.58, 44.20)^***^ = 1.65  *p* = 0.21  partial η^2^  = 0.06 | *F* (1.58, 44.20)^⬩^ = 3.23  *p* = 0.057  partial η^2^  = 0.11 | ***F* (1,28) = 8.86**  ***p* = 0.006**  partial η^2^  = 0.24 |
| *IL-12p70* | 0.09±0.02 | 0.06±0.01 | 0.13±0.05 | 0.23±0.07 | 0.13±0.05 | 0.10±0.04 | *F (*2,36) = 1.22  *p* = 0.31  partial η^2^  = 0.06 | *F (*2,36) = 2.31  *p* = 0.11  partial η^2^  = 0.11 | *F* (1,18) = 2.20  *p* = 0.16  partial η^2^  = 0.11 |
| *IL-13* | 0.33±0.08 | 0.31±0.09 | 0.30±0.08 | 0.23±0.08 | 0.29±0.06 | 0.37±0.10 | *F* (2, 60) = 0.86  *p* = 0.43  partial η^2^  = 0.028 | *F* (2, 60) = 1.81  *p* = 0.17  partial η^2^  = 0.06 | *F* (1,30) = 0.01  *p* = 0.92  partial η^2^  = 0.00 |
| *IL-17A* | 1.82±0.51 | 2.53±0.53 | 2.94±0.62 | 1.61±0.32 | .301±0.67 | 2.57±0.45 | *F* (1.56, 48.32)**^•^**= 7.37  *p* = 0.003  partial η2 = 0.19 | *F* (1.56, 48.32)**^•^**= 1.02  *p* = 0.35  partial η2 = 0.03 | *F* (1,31) = 0.002  *p* = 0.96  partial η^2^  = 0.00 |
| *TNF-α* | 4.17±0.43 | 5.82±0.52 | 6.33±0.64 | 4.90±0.65 | 6.56±0.88 | 8.31±1.24 | ***F* (1.45, 46.24)^◼^ 22.09**  ***p*<0.001**  partial η^2^  = 0.41 | *F* (1.45, 46.24)^◼^ = 1.46  *p*=0.24  partial η^2^  = 0.04 | *F* (1,32) = 1.70  *p* = 0.20  partial η^2^  = 0.05 |
| *VEGF* | 200.19±36.31 | 175.83±30.98 | 158.19±25.41 | 217.17±52.81 | 201.97±55.08 | 182.64±46.99 | *F* (1.58, 42.53)^⬩^= 2.87  *p* = 0.065  partial η^2^  = 0.10 | *F* (1.58, 42.53)^⬩^ = 0.05  *p* = 0.95  partial η^2^  = 0.02 | *F* (1,27) = 0.18  *p* = 0.68  partial η^2^  = 0.006 |

***Notes****:* ***bold*** *denotes significant test result p > 0.05; underlined denotes statistical trend; where indicated, Greenhouse-Geisser correction applied to df - * ε = 0.69;* ***•****0.78; ⬩0.79;* ***^◼^****0.72;* *HCV – Hepatitis C Viral infection; TW – Treatment Week; IFN – interferon; IL – Interleukin; TNF – Tumour Necrosis Factor; VEGF – Vascular Endothelial Growth Factor*

***Supplementary Table 5: Changes in kynurenine pathway metabolites during IFN-α treatment in Persistent Fatigue vs Resolved Fatigue patients (repeated measures GLM)***

| Marker | HCV RF | | | HCV PF | | | Statistics | | |
| --- | --- | --- | --- | --- | --- | --- | --- | --- | --- |
|  | TW0 | TW4 | TW24 | TW0 | TW4 | TW24 | Time | Time X Group | Group |
| *3-HK* | 11.5±1.04 | 13.12±1.28 | 14.19±1.43 | 11.32±1.52 | 11.7±1.93 | 14.84±3.09 | ***F* (2,68) = 3.86**  ***p* = 0.03**  **partial η^2^ = 0.10** | *F* (2,68) = 0.41  *p* =0.66  partial η^2^ = 0.01 | *F* (1,34) = 0.03  *p* = 0.88  partial η^2^ = 0.01 |
| *3-HK/Kyn* | 2.99±0.24 | 2.89±0.25 | 3.13±0.28 | 3.01±0.33 | 3.14±0.54 | 3.49±0.51 | *F* (2,68) = 1.50  *p* = 0.23  partial η^2^ = 0.04 | *F* (2,68) = 0.34  *p* = 0.71  partial η^2^ = 0.01 | *F* (1,34) = 0.23  *p* = 0.64  partial η^2^ = 0.007 |
| *Kyn* | 391.37±23.62 | 471.31±35.57 | 458.43±33.93 | 373.98±25.85 | 382.8±27.41 | 402.58±34.61 | ***F* (2,66) = 3.81**  ***p* = 0.03**  partial η2 = 0.11 | *F* (2,66) = 1.61  *p* = 0.21  partial η^2^ = 0.05 | *F* (1,33) = 0.84  *p* = 0.37  partial η^2^ = 0.03 |
| *Kyn/Trp ratio* | 2.18±0.12 | 2.82±0.2 | 2.72±0.17 | 2.19±0.14 | 2.34±0.19 | 2.67±0.24 | ***F* (2,68) = 11.22**  ***p*<0.001**  **partial η^2^ = 0.25** | *F* (2,68) = 2.71  *p*=0.08  partial η^2^ = 0.07 | *F* (1,34) = 0.48  *p*=0.49  partial η^2^ = 0.01 |
| *Kynurenic acid* | 8.26±0.73 | 7.81±0.66 | 7.44±0.63 | 7.54±0.68 | 7.30±0.70 | 6.00±0.73 | ***F* (2,66) = 4.57**  ***p* = 0.014**  **partial η^2^ = 0.12** | *F* (2,66) = 0.74  *p* = 0.48  partial η^2^ = 0.02 | *F* (1,33) = 0.80  *p* = 0.38  partial η^2^ = 0.02 |
| *Pic acid* | 78.54±6.99 | 72.02±5.13 | 71.85±5.59 | 71.94±10.06 | 61.88±8.41 | 62.9±9.5 | *F*  (2, 68) = 2.49  *p* = 0.091  partial η^2^ = 0.07 | *F*  (2, 68) = 0.09  *p* = 0.91  partial η^2^ = 0.003 | *F* (1,34) = 0.77  *p* = 0.39  partial η^2^ = 0.02 |
| *Quinaldic acid* | 2.2±0.25 | 1.69±0.15 | 1.47±0.16 | 2.13±0.39 | 1.93±0.28 | 1.49±0.3 | ***F* (2,68) = 6.62**  ***p* = 0.002**  **partial η^2^ = 0.16** | *F* (2,68) = 0.37  *p* = 0.69  partial η^2^ = 0.01 | *F* (1,34) = 0.05  *p* = 0.82  partial η^2^ = 0.002 |
| *Quinolinic acid* | 53.18±5.93 | 58.66±5.46 | 55.15±5.38 | 48.72±5.57 | 45.79±5.98 | 58.89±8.83 | *F*  (2, 68) = 1.37  *p* = 0.26  partial η^2^ = 0.07 | *F*  (2, 68) = 2.31  *p* = 0.11  partial η^2^ = 0.06 | *F*  (1,34) = 0.28  *p* = 0.60  partial η^2^ = 0.008 |
| *Tryptophan* | 18121.36±639.18 | 16980.13±778.27 | 17129.5±836.54 | 17309.95±834.3 | 16843.21±1100.54 | 15530.26±1204.52 | *F* (1.71, 57.98)***= 2.59  *p* = 0.092  partial η^2^ = 0.07 | *F* (1.71, 57.98)***= 0.72  *p* = 0.47  partial η^2^ = 0.02 | *F*  (1,34) = 0.56  *p* = 0.46  partial η^2^ = 0.02 |
| *Xanthurenic acid* | 3.66±0.51 | 3.33±0.44 | 3.32±0.47 | 2.46±0.35 | 3.29±0.87 | 2.09±0.48 | *F*  (2, 66) = 1.10  *p* = 0.34  partial η^2^ = 0.03 | *F*  (2, 66) = 1.29  *p* = 0.28  partial η^2^ = 0.04 | *F* (1,33) = 1.32  *p* = 0.26  partial η^2^ = 0.04 |

***Notes****:* ***bold*** *denotes significant test result p > 0.05; underlined denotes statistical trend; where indicated, Greenhouse-Geisser correction applied to df - *ε = 0.85; HCV – Hepatitis C Viral infection; TW – Treatment Week; HK – Hydroxykynurenine; Kyn – Kynurenine; Trp – Tryptophan; Pic – Picolinic*
